# Editorial: Extracellular Vesicles as Immunomodulatory Mediators in Inflammatory Processes

**DOI:** 10.3389/fimmu.2022.867324

**Published:** 2022-03-03

**Authors:** Paula Barbim Donate, Yann Lamarre, Karina Pino-Lagos, Danièle Noël, Fausto Almeida

**Affiliations:** ^1^ Department of Pharmacology, Ribeirao Preto Medical School, University of Sao Paulo, Ribeirão Preto, Brazil; ^2^ Center for Cell-Based Therapy of the Regional Blood Center of Ribeirão Preto, Ribeirão Preto, Brazil; ^3^ Centro de Investigación Biomédica, Facultad de Medicina, Universidad de los Andes, Las Condes, Chile; ^4^ IRMB, Univ Montpellier, INSERM, CHU Montpellier, Montpellier, France; ^5^ Department of Biochemistry and Immunology, Ribeirao Preto Medical School, University of Sao Paulo, Ribeirao Preto, Brazil

**Keywords:** extracellular vesicles, inflammation, immune response, cell-cell communication, regulation

Among the plurality of signaling cascades, the communication between cells represents a central component in inflammatory processes. Extracellular vesicles (EVs), a recognized physiological mechanism of cell-cell communication, carry and protect important biological information that shapes immune responses. The roles of cell-derived EVs start to be unraveled over recent years, both in physiological and pathological conditions. To understand how extracellular vesicles (EVs) modulate the immune system is crucial to achieve new therapeutic and prophylactic strategies for inflammatory conditions.

This Research Topic presents 13 themed articles (8 reviews and 5 original research articles) that summarize some recent advances and provide new facts improving our knowledge of the complex biology of how EVs modulate the immune response. For example, Santos and Almeida, who also edits this Research Topic, summarized important studies from a historical perspective, regarding current investigations on EV-based vaccines. The authors discussed several results obtained from completed clinical trials that have used EV-based vaccines in the treatment of cancer and coronavirus disease. Sun et al., provided an overview of the studies related to EVs in the immune system during *Mycobacterium tuberculosis* infection, and have addressed the use of EVs for vaccines and therapies against tuberculosis. Reis et al. identified the composition of small molecules in EVs produced by *Cryptococcus gattii*, and their improved effect on the survival of *Galleria mellonella* infected with *C. gatti* or *Cryptococcus neoformans*.

Different cell types release distinct repertoire of EVs. In this sense, Hovhannisyan et al. highlighted the contribution of EVs produced in stroma and epithelia, by cells as keratinocytes, hepatocytes, and fibroblast in modulating the immune response, and summarized the relevant studies regarding the role of non-immune cell-derived EVs in some mechanisms of allergy.

In contrast to their role in the pathogenesis of inflammatory conditions, EVs have demonstrated the potential to attenuate inflammatory disorders, and are being explored as therapeutic agents. Mesenchymal stromal cells (MSC)-derived EVs have gained significant interest regarding their immunoregulatory and immunosuppressive effects in clinical therapy. In this Research Topic, several groups have addressed MSC-derived EVs. Shen et al. summarized the effect of MSC-derived EVs on the immune system and their potential use in autoimmune diseases. On the other hand, Valade et al. described the potential role of MSC-derived EVs on the pathophysiological environment of traumatic hemorrhagic shock, emphasizing the impact of the crosstalk between the immune system and organs on their functions. Additionally, Crawford et al. discussed the current knowledge of EVs derived from MSCs and their miRNA cargo on target cells associated with the pathology of rheumatoid arthritis and osteoarthritis, as well, their potential therapeutic impact in these two rheumatic diseases.

In the work by Merino et al., we learned that membrane particles derived from adipose tissue-derived MSCs play regenerative effects on endothelial cells, opening new perspectives for vascular diseases treatment where inflammatory processes damage the endothelium. Still studying MSCs, Gonçalves et al. have expanded our knowledge on the mechanisms involved in the uptake and cellular functions of MSC-derived membrane particles by macrophages and human umbilical vein endothelial cells. Elashiry et al. provided knowledge on the protein cargo identification of dendritic cell-derived EVs and their distribution in different tissues.

It is also well recognized that EV contents are altered depending on the local microenvironment, supporting the possibility of their use as biomarkers due to their stability in body fluids. Bai et al., highlighted the studies using placenta-derived EVs on immune cells, particularly showing their key role of exosomes as modulators of maternal immune tolerance during pregnancy. Aarts et al. discussed the immunomodulating capacity of milk-derived EVs and their potential application in immunotherapies for systemic inflammatory diseases. Finally, Carvajal et al. demonstrated that lipocalins and the specific urinary miR-21-5p and Let-7i-5p contained in EVs are potential biomarkers of primary aldosteronism.

In summary, this Research Topic improves our comprehension of how EVs affect the inflammatory processes, describing potential targets for immunotherapies and mechanisms by which EVs modulate the immune response, highlighting the importance of new studies to elucidate the biology of EVs. Despite the numerous remaining challenges in understanding EV biology, it represents an active area of research that brings new perspectives for the future of therapeutic applications to treat chronic inflammation-based diseases.

## Authors Contributions

PD and FA drafted the Editorial, while YL, KP-L, and DN contributed to editing. All authors conceived and designed the work and provided final approval of the version to be published.

## Conflict of Interest

The authors declare that the research was conducted in the absence of any commercial or financial relationships that could be construed as a potential conflict of interest.

## Publisher’s Note

All claims expressed in this article are solely those of the authors and do not necessarily represent those of their affiliated organizations, or those of the publisher, the editors and the reviewers. Any product that may be evaluated in this article, or claim that may be made by its manufacturer, is not guaranteed or endorsed by the publisher.

